# 867. Telehealth and HIV Care During the COVID-19 Pandemic

**DOI:** 10.1093/ofid/ofab466.1062

**Published:** 2021-12-04

**Authors:** Smitha Gudipati, Monica Lee, Indira Brar, Norman Markowitz

**Affiliations:** 1 Henry Ford Health System, Detroit, Michigan; 2 Henry Ford Hospital, detroit, Michigan

## Abstract

**Background:**

The COVID-19 Pandemic led to many restrictions in health care services, and as a consequence, an expansion of telehealth capabilities. In order to meet the needs of PLWH along the Care Continuum, we developed a process to promote the use of our MyChart app. This HIPAA-compliant app allows patients to view their medical records, communicate with their providers, make appointments, and have video visits on their smart devices. This report describes our preliminary findings.

**Methods:**

PLWH enrolled in the Ryan White Program, in the Infectious Diseases Clinic at Henry Ford Hospital who had not used telehealth services were asked to sign up for our MyChart (electronic medical record software) initiative. A telehealth Navigator interviewed and taught PLWH how to download and use MyChart, and supplied pre-loaded phones, as needed, to make virtual visits accessible. We collected demographic and clinical information and reasons for not using telehealth services.

**Results:**

From October 2020 to May 2021, 209 PLWH were enrolled into our pilot program (Table 1). Of these: 48% were 45-64 years old (yo), while 21% were >/+ 60 yo and 3% < 25 yo; 75% were male, 85% Black; 48% MSM, and 84% virally suppressed (HIV RNA < 200 copies/mm^3^). When asked why they were not using telehealth services, 29% reported a lack of technology or capability to install MyChart on their phones, 27% needed further education, and 18% and had not prioritized installation of the application.

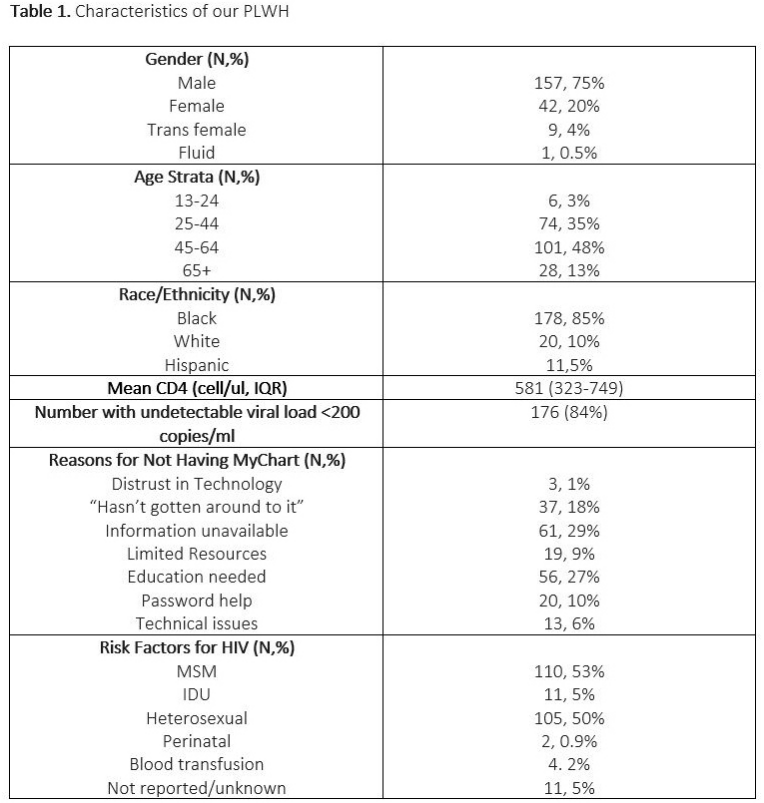

**Conclusion:**

The crises created by the COVID-19 pandemic revealed a new role for telehealth services. Although available to all PLWH in our RW program, many had never used telehealth services. Over half lacked compatible devices or needed help to download or use the app. Compared to younger PLWH, older individuals were more likely to need assistance. Further work is needed to understand and promote digital parity.

**Disclosures:**

**All Authors**: No reported disclosures

